# Thiostrepton interacts covalently with Rpt subunits of the 19S proteasome and proteasome substrates

**DOI:** 10.1111/jcmm.12602

**Published:** 2015-05-30

**Authors:** Cristinel Sandu, Nagaranjan Chandramouli, Joseph Fraser Glickman, Henrik Molina, Chueh-Ling Kuo, Nikolay Kukushkin, Alfred L Goldberg, Hermann Steller

**Affiliations:** a Howard Hughes Medical Institute, Strang Laboratory of Apoptosis and Cancer Biology, The Rockefeller UniversityNew York, NY, USA; b Proteomics Resource Center, The Rockefeller UniversityNew York, NY, USA; c High Throughput Screening Resource Center, The Rockefeller UniversityNew York, NY, USA; d Department of Cell Biology, Harvard Medical SchoolBoston, MA, USA

**Keywords:** thiol, protein degradation, protein chemical modification, proteasome, ubiquitin

## Abstract

Here, we report a novel mechanism of proteasome inhibition mediated by Thiostrepton (Thsp), which interacts covalently with Rpt subunits of the 19S proteasome and proteasome substrates. We identified Thsp in a cell-based high-throughput screen using a fluorescent reporter sensitive to degradation by the ubiquitin–proteasome pathway. Thiostrepton behaves as a proteasome inhibitor in several paradigms, including cell-based reporters, detection of global ubiquitination status, and proteasome-mediated labile protein degradation. *In vitro*, Thsp does not block the chymotrypsin activity of the 26S proteasome. In a cell-based IκBα degradation assay, Thsp is a slow inhibitor and 4 hrs of treatment achieves the same effects as MG-132 at 30 min. We show that Thsp forms covalent adducts with proteins in human cells and demonstrate their nature by mass spectrometry. Furthermore, the ability of Thsp to interact covalently with the cysteine residues is essential for its proteasome inhibitory function. We further show that a Thsp modified peptide cannot be degraded by proteasomes *in vitro*. Importantly, we demonstrate that Thsp binds covalently to Rpt subunits of the 19S regulatory particle and forms bridges with a proteasome substrate. Taken together, our results uncover an important role of Thsp in 19S proteasome inhibition.

## Introduction

We identified Thiostrepton (Thsp) as a strong hit in a cell-based screen for modulators of *Drosophila* Inhibitor of Apoptosis Protein 1 (DIAP1) stability in human cells. Inhibitor of apoptosis proteins (IAPs), including DIAP1 are E3 ubiquitin ligases that regulate the levels of important proapoptotic factors such as caspases, by binding and targeting them for degradation by the ubiquitin -proteasome pathway (UPP) 1–3.

Thiostrepton is a natural antibiotic produced by microorganisms of the *Streptomyces* genus 4–6. It is a large molecule (1.66 kD), translated by the ribosome and following leader sequence cleavage it undergoes extensive post-translational modifications 7. In bacteria, Thsp blocks protein translation through binding to the GTPase centre of the 70S ribosome, in a cleft between L11 subunit and H43/H44 of the 23S rRNA, and in this way obstructs the recruitment and turnover of the elongation factor EF-G 8–10. In mammals, Thsp does not block the cytoplasmic protein translation, because of the sequence difference in 28/23S rRNA that prevents Thsp binding 11. Reminiscent of its function in bacteria, Thsp was shown, however, to inhibit mammalian mitochondrial translation 12. Consistent with these observations it was shown that Thsp reduces the levels of mitochondrial cytochrome oxidase I, triggers reactive oxigen species (ROS) (in combination with arsenic trioxide) where this effect can be rescued by free radical scavenger *N*-acetyl-l-cysteine (NAC) 13.

Using gene expression profiling, it was shown that Thsp induces oxidative and proteotoxic stress, as revealed by the transcriptional up-regulation of heat shock, oxidative stress and endoplasmic reticulum (ER) stress genes 14. Oxidative and proteotoxic stress effects were relieved by treatment with the anti-oxidant NAC. Using a mass spectrometry approach, we also showed that proteasome inhibitor MG-132 and Thsp induce up-regulation of heat shock proteins, consistent with a proteotoxic stress response 15. Recently, it was suggested that the mechanism of oxidative stress induced by Thsp is caused by the inactivation of mitochondrial peroxiredoxin-3, thus disabling an important anti-oxidative network 16. Thiostrepton triggered a protein mobility shift change in peroxiredoxin-3, and it was proposed that this is caused by a covalent adduct formation, similar to the interaction between Thsp and TipAS protein from *Streptomyces lividans* 17. Thiostrepton was also shown to inhibit FoxM1 function 18–21.

It has been reported that Thsp acts as an inhibitor of the 20S proteasome chymotrypsin activity 22,23. However, the mechanism by which Thsp inhibits proteasome activity remains unknown. The ability of Thsp to inhibit the proteasome is of potential clinical relevance as the proteasome is the major cellular protein degradation factory and proteasome inhibitors are currently used for human therapy (Bortezomib/Velcade) or are in clinical trials 24,25. Bortezomib is used for the treatment of relapsed multiple myeloma and mantle cell lymphoma. Identification of novel proteasome inhibitors may provide alternatives to current therapy that are potentially less toxic or target the proteasome by a distinct mechanism.

Thiostrepton induces apoptotic cell death in various human cancers, including breast, melanoma, leukaemia, liver cancer or malignant mesothelioma 14,16,19,20,26,27. Cancer cells appear to be more sensitive to Thsp than normal cells, as it was shown for melanoma cells *versus* primary melanocytes 14,15. Moreover, although Thsp induces proteotoxic stress in both melanoma and primary melanocytes, only cancer cells undergo cell death 15. Thiostrepton was not considered for human therapy because of its poor solubility and unfavourable pharmacodynamics. However, because of its significant anti-cancer properties and with increasing clarification of its mechanisms of action, Thsp remains an interesting molecule that may have possible clinical utility. Currently, Thsp is used in mammals as topical medication in veterinary medicine for the treatment of mastitis and dermatological disorders 28.

In this study, we found that Thsp acts as an inhibitor of the 19S proteasome. Thiostrepton forms adducts with human proteins and its ability to interact covalently with cysteine residues is essential for proteasome inhibition. We characterized the nature of the adducts and show that Thsp bridges between Rpt proteasome subunits and proteasome substrates. These findings suggest a novel mode of proteasome inhibition, which occurs at the substrate unfolding/translocation step.

## Materials and methods

### Cell culture, transfections and plasmids

#### DIAP1 sensor cell line

HEK293 cells were stably cotransfected with pcDNA3.1(+)Puro-DIAP1ΔR-YFP (DIAP1 corresponding to residues 1–320 fused to YFP) construct and pcDNA3.1(+)Puro-Rpr-HA 29. Sensor cells were established from a single cell that was resistant to Puromycin treatment, following an established procedure 30. Dual-colour DIAP1 sensor cells were generated by stable cotransfection of HEK293 with pcDNA3.1(+)Puro-DIAP1ΔR-mCherry (DIAP1 corresponding to residues 1–320 fused to mCherry gene) and Rpr-HA construct cloned in pIRES2-EGFP vector (Invitrogen, Carlsbad, CA, USA). Thiostrepton EC_50_ was determined in using this dual -colour DIAP1 sensor cell line. Increasing amounts of Thsp (0–20 μM) was incubated with 3000 cells in a 40 μl culture volume (384 well plates) for 18 hrs. The plates were scanned using ImageXpress Velos Laser Scanning Cytometer (Molecular Devices, Sunnyvale, CA, USA) to collect 5-μm resolution red and green fluorescence images. The images were segmented using the ImageXpress Velos analysis software (Molecular Devices) to recognize individual fluorescent particles on both channels. The data for each concentration were represented as total fluorescence (TF) red/TF green*100. For screening purposes this fluorescence number above was normalized against the fluorescence number of dimethyl sulfoxide (DMSO) (0%) and that of 10 μM MG-132 (100%). The proteasome sensor consists of HEK293 cells transfected with pZsProSensor-1 plasmid (Clontech, Palo Alto, CA, USA). Positive colonies were selected based on detectible green fluorescence.

### Proteins, compounds, antibodies

Rpr protein (residues 1–65) followed by GSSHHHHHH tag was purified as described previously 29. RprPep (AVAFYIPDYPYDVVPDYATSCHPKTGRKSGKYRKPSQ), at 95% purity was synthesized by (ELIM Bio, Hayward, CA, USA). All the compounds in this work, otherwise specified, were dissolved in DMSO. Compounds were purchased from commercial inventories as follows: MG-132 (Calbiochem, San Diego, CA, USA), Thsp (Tocris Cookson Inc. (Ellisville, MO, USA)). Comp-3 was kindly provided by Dr. Patrick G. Harran, UCLA or synthesized by Ouathek Ouerfelli and Barney Yoo at the Organic Synthesis Core Facility of the MSKCC. The antibodies used in this work were purchased as follows: rabbit anti-DIAP1 (laboratory collection), chicken anti-Rpr (laboratory collection), rabbit anti-GFP (Santa Cruz Biotechnology, Inc., Santa Cruz, CA, USA), rabbit anti-Ups24 (Protein Group Inc., Chicago, IL, USA), anti-HA-HRP (Roche, Indianapolis, IN, USA), mouse anti-polyubiquitin, FK2 clone (Millipore, Billerica, MA, USA), mouse anti-p53 (Santa Cruz Biotechnology, Inc), rabbit anti-IκBα (Santa Cruz Biotechnology, Inc), mouse anti-p21 (Cell Signalling Technology, Danvers, MA, USA), rabbit anti-Mcl1 (Cell Signalling), mouse anti-GAPDH (Abcam, Cambridge, MA, USA), anti-Rpt1 to anti-Rpt6 (Enzo Life Sciences, Farmingdale, NY, USA). Purified bovine 26S proteasome (UBP Bio, Aurora, Co, USA), purified rabbit 20S proteasome (Boston Biochem, Cambridge, MA, USA), human and bovine 19S regulatory particle (UBP Bio).

### Western blot and immunoprecipitation of DIAP1 complexes

Cells were lysed in lysis buffer (10 mM Tris, pH 8.0, 100 mM NaCl, 0.5% NP-40) and extracts were cleared by centrifugation at 20,817 × *g*. 15 μg cell extract were regularly used for detection of proteins by WB. Immunoprecipitation of DIAP1ΔR-YFP complexes from extracts derived from DIAP1 sensor cells were achieved using a GFP purification kit (Miltenyi Biotech, San Diego, CA, USA), following manufacturer instructions.

### Proteasome activity determination

Proteasome activities were determined using purified bovine 20S proteasome (Boston Biochem) or purified bovine 26S proteasome (UBP Bio) and using synthetic proteasome substrates. The three specific activities (chymotrypsin-, trypsin- and caspase-like) of the proteasome were determined using either fluorogenic tri-peptide substrates (Bachem, Torrance, CA, USA) or a chemiluminiscent Protesome-Glo™ chemotrypsin-like assay (Promega, Madison, WI, USA). A typical reaction consisted of 1.3 nM purified proteasome, 20 μM substrate in a volume of 40 μl. The compounds were added to the reaction at a 5 or 10 μM final concentration. The reaction is performed in a white 96 well plate with clear bottom. The hydrolysis of fluorescent peptides was monitored continuously for 30 min. and the rates of hydrolysis were shown in the figures. The chemi-luminescence reactions were measured using a Spectramax M2 plate reader (Molecular Devices). A time-dependent kinetic was performed for 30 min. (10 reads every other 3 min.) and data were analysed using Softmax Pro 4.7.1. (Molecular Devices). Proteasome activity is represented as Vmax, calculated from the slope (RTL/sec.) of the reaction.

### *In vitro* assay for Rpr-Thsp adduct formation

The assay consisted of 10 μl reaction containing either 350 pmol Rpr and 2.5 nmol Thsp or 350 pmol Rpr and 2.5 nmol Thsp and 7.5 nmol L-cysteine. The reaction buffer was 25 mM Tris, pH 8.0, 50 mM NaCl. The reaction was incubated at 37°C for 0–3 hrs. After the determined time, the reactions were analysed by SDS-PAGE, WB or by mass spectrometry. Formation of RprPep-Thsp adduct was done similarly in a 10 μl reaction consisting of 2.38 nmol RprPep and 5 nmol Thsp in 25 mM Tris, pH 8.0, 50 mM NaCl. The reaction was incubated for 3 hrs at RT and the formation of RprPep-Thsp was validated by SDS-PAGE.

### *In vitro* ubiquitination of RprPep and RprPep-Thsp adducts

1.1 nmol of RprPep or RprPep-Thsp adduct (prepared as above), was incubated with 1.0 μg E1, 1.0 μg E2, 1.0 μg Flag-DIAP1 and 3 μg Ubiquitin in 20 μl reaction. The reaction buffer was 25 mM Tris, pH 7.5, 50 mM NaCl, 4 mM ATP and 5 mM MgCl. The reaction was incubated for 100 min. at RT, and flash frozen until further use.

### *In vitro* degradation of RprPep by purified 26S proteasome

Degradation reactions consisted of 0, 0.4, 1.0 and 2.0 pmol bovine 26S proteasome (UBP Bio) and 55 pmol ubiquitinated RprPep or RprPep-Thsp adducts in a final volume of 10 μl. The reaction buffer was 25 mM Tris, pH 7.5, 50 mM NaCl, 4 mM ATP and 5 mM MgCl, 250 μM DTT. Following incubation at RT for 40 min., the reactions were stopped by the addition of 5 μl sample buffer and subsequent boiling at 95°C. The samples were separated by SDS-PAGE, and RprPep and RprPep-Thsp adduct deubiquitination or degradation were monitored by WB using an ubiquitin antibody (FK2) or Rpr antibody.

### Mass spectrometry analysis of Rpr complexes

Matrix Assisted Laser Desorption Ionization Time-of-Flight (MALDI-ToF) mass spectrometry analysis (Voyager-DE-STR; Applied Biosystems, Carlsbad, CA, USA) was performed in linear mode using a standard Nitrogen laser. Recrystallized alpha-cyano-4-hydroxycinnamic 31 (Sigma-Aldrich, Steinheim, Germany) was used as MALDI matrix. Samples of Rpr (0.3 μg/μl) alone or reacted with Thsp, respectively, were each diluted 1:1 with formic acid (‘High Purity Grade’, Sigma-Aldrich). One microlitre of each sample was spotted on to a standard stainless steel MALDI target followed by the addition of 2 μl matrix solution. The spotted solution was gently mixed with the tip of a pipette for better mixing and crystallization. Mass spectra were acquired using an acceleration voltage of 20 kV and an extraction delay of 200–1200 nsec. For each spot, spectra from 500 laser shots were summed. All measurements were performed in replicate.

### Molecular structure visualization and distance measurements

Distance measurements, structural images rendering and snap-shots acquisition of Thsp (PDB: 2L2W), yeast 26S proteasome (PDB: 4B4T) or archaeal proteasome (PDB: 3IPM) were achieved using molecular visualization software Pymol Software (DeLano Scientific LLC, Palo Alto, CA, USA).

## Results

### Thiostrepton stabilizes a labile DIAP1-based protein sensor in human cells

**Figure 1:**
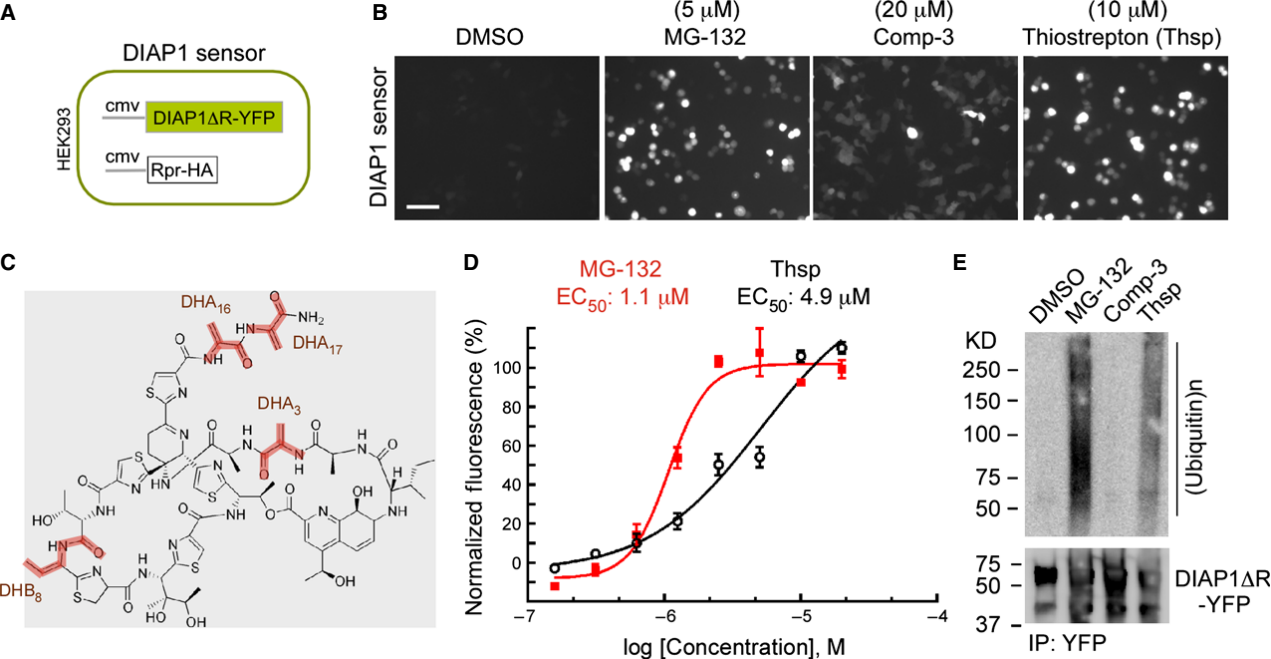
Thiostrepton induces accumulation of a labile reporter in human cells. (A) Schematic representation of a fluorescence-based DIAP1-sensor, represented by HEK293 cells stably cotransfected with DIAP1ΔR-YFP fusion and Rpr-HA. IAP-antagonist Rpr binds to DIAP1 and triggers its ubiquitination and degradation. (B) Fluorescence micrographs of DIAP1-sensor cells, showing DIAP1ΔR-YFP fluorescence response following DMSO, MG-132, Comp-3 (synthetic IAP-antagonist) and Thsp treatment for 18 hrs. (C) Chemical structure of Thsp with highlighted reactive dehydroalanine residues (DHA_3_/DHA_16_/DHA_17_) and the dehydrobutyrine residue (DHB_8_). (D) Diagram showing the determination of EC_50_ for MG-132 and Thsp in DIAP1-sensor cells. (E) Thsp triggers accumulation of polyubiquitinated DIAP1 species. DIAP1ΔR-YFP was immunoprecipitated from IAP-sensor cells treated with the specified compounds, and the ubiquitination status and DIAP1 levels were determined by WB with anti-ubiquitin and anti-DIAP1 antibodies.

We identified Thsp in a high-content cell-based small molecule screen for modulators of DIAP1 stability in human cells. The IAP-antagonist Rpr was previously shown to bind DIAP1 and stimulate its self-ubiquitination and degradation by the UPP 29,32,33. Furthermore, Rpr can stimulate degradation of DIAP1 lacking the E3 ligase activity (DIAP1ΔR) in human cells 29. On the basis of these observations, we designed a fluorescent reporter cell line that is sensitive to proteasome inhibition. Specifically, we generated HEK293 cells stably transfected with both fluorescent DIAP1ΔR and Rpr-HA (Fig.1A). Using this line, we performed a small-molecule screen for modulators of DIAP1ΔR degradation. Chymotrypsin proteasome inhibitor MG-132 and a peptido-mimetic IAP-antagonist (Comp-3) caused a fluorescence increase in this assay and were used as positive controls 34. Thiostrepton showed a strong activity in the sensor cells and increased DIAP1ΔR-YFP fluorescence very similar to MG-132 (Fig.1B). Thiostrepton is a known bioactive natural compound that is ribosomally produced by Gram-positive bacteria of the genus *Streptomyces*, and which undergo extensive post-translational modifications 7. Thiostrepton contains four reactive groups, three dehydroalanine (DHA) and one dehydrobutyrine (DHB), which can interact covalently with Cysteine residues, as it was demonstrated with *Streptomyces* TipAS protein 17 (Fig.1C). We have quantified Thsp activity and found that it is a potent compound with an EC_50_ of 4.9 μM in DIAP1 sensor cells, slightly weaker than MG-132 with an EC_50_ of 1.1 μM (Fig.1D). To understand the mechanism of Thsp-induced DIAP1ΔR-YFP fluorescence increase, we determined DIAP1ΔR ubiquitination status in IP fractions from DIAP1 sensor cells treated with compounds (Fig.1E). Interestingly, Thsp induces accumulation of polyubiquitinated DIAP1ΔR, similar to MG-132 but distinct from Comp-3, an effect that is consistent with a role of Thsp in UPP inhibition.

### Thiostrepton blocks proteasome function in human cells

**Figure 2:**
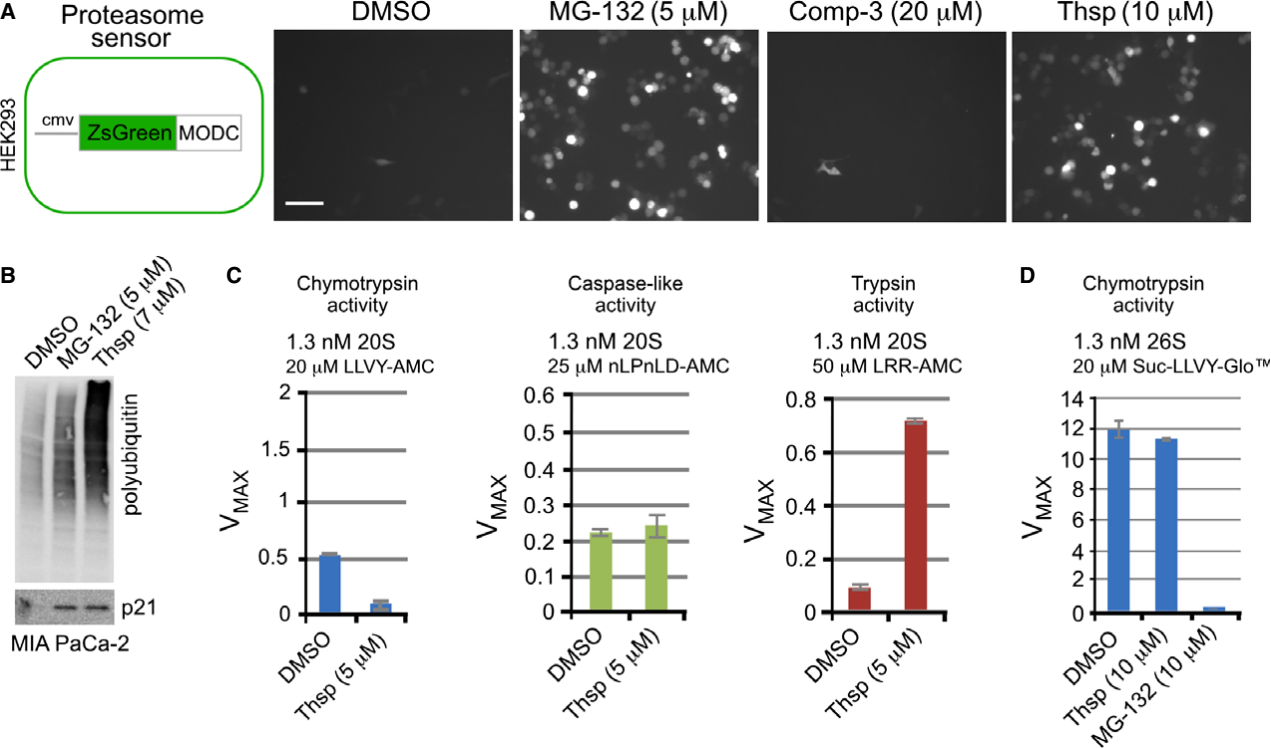
Effect of Thiostrepton on proteasome activity in human cells and *in vitro*. (A) Left, schematic representation of the ZsGreen-MODC sensor cell line. Human HEK293 cells were stably transfected with a construct that entails *Zoanthus sp*. GFP gene fused to mouse ornithine decarboxylase degron (MODC). Right, microscopy images showing the fluorescence of the labile GFP-MODC reporter following DMSO, MG-132, Comp-3 and Thsp treatment for 21 hrs. (B) WB detection of polyubiquitin species and p21 accumulation in extracts of MIA-PaCa-2 cells treated with DMSO, MG-132 and Thsp for 20 hrs. (C) Effect of Thsp on catalytic activities (chymotrypsin-, trypsin- and caspase-like) of the purified bovine liver 20S proteasome. (D) Effect of Thsp and MG-132 on the activity of purified bovine 26S proteasome.

To further understand the role of Thsp in UPP inhibition, we have tested whether Thsp can signal in a proteasome sensor cell line (Fig.2A). The sensor is based on a fluorescent and unstable chimeric protein (ZsGreen-MODC) that is reportedly degraded by the proteasome 35–37. The sensor is functional and specific as it is responsive to a proteasome inhibitor (MG-132) but not to IAP-antagonist Comp-3 (Fig.2A). Thiostrepton stimulates accumulation of fluorescence in these cells, indicating that it plays a role in proteasome inhibition (Fig.2A). Like MG-132, Thsp triggers accumulation of polyubiquitinated species and labile endogenous p21 in MIA-PaCa 2 cells (Fig.2B), further supporting a role in proteasome inhibition and is consistent with previous reports 14,22. Next, we tested the ability of Thsp to block the three catalytic activities in the context of purified 20S proteasome, using small synthetic-labelled substrates (Fig.2C). Thiostrepton decreases the chymotrypsin activity (hydrophobic substrates) of the 20S proteasome, while the caspase activity (acidic substrates) is not affected. Surprisingly, Thsp appears to increase the trypsin-like activity (basic substrates) of the 20S proteasome by four-fold. We also tested the effect of Thsp on the chymotrypsin activity in the context of purified 26S proteasome (Fig.2D). Interestingly, Thsp does not block the chymotrypsin activity in 26S proteasome, which is at odds with the observations involving 20S proteasome. As a control, chymotrypsin activity inhibitor MG-132 blocks activity in this assay very efficiently. The overall chymotrypsin activity of 26S proteasome is much higher than that of 20S proteasome, which is explained by the difference in the state of 20S gate opening between these proteasome species 38. Thiostrepton was previously reported to inhibit the chymotrypsin activity of the proteasome 22,23. However, our *in vitro* assays using purified 20S and 26S proteasomes revealed that the effects are more complex.

### Thiostrepton can bind covalently to proteins in human cells

**Figure 3:**
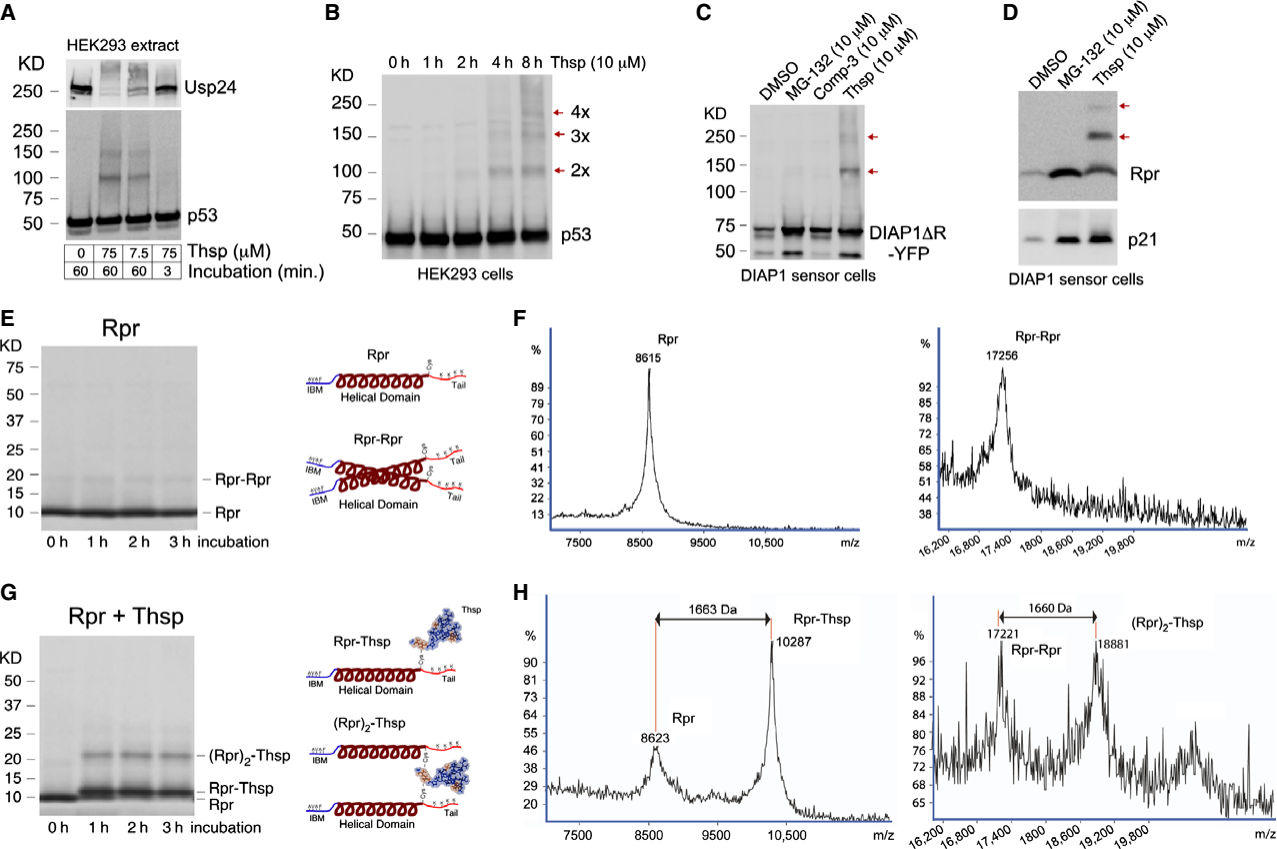
Thiostrepton interacts covalently with proteins in human cells. (A) WB detection of Usp24 and p53 in HEK293 cell extracts incubated with different concentrations of Thsp, for different times. Note the formation of p53 and Usp24 dimers/multimers, an effect that is dependent on Thsp concentration and incubation time. (B) Time-dependent formation of p53 multimers in HEK293 cells treated with Thsp (10 μM), as detected by WB. (C) WB detection of DIAP1ΔR-YFP in DIAP1-sensor cells treated with DMSO, MG-132, IAP-antagonist (Comp-3) and Thsp for 21 hrs. Besides DIAP1ΔR-YFP accumulation, Thsp also triggers formation of DIAP1 dimers and multimers. (D) WB detection of Rpr-HA and p21 in DIAP1-sensor cells treated with DMSO, MG-132 and Thsp for 21 hrs. Thiostrepton -dependent formation of Rpr dimers/multimers becomes apparent. (E) Left, Coomassie-stained SDS-PAGE gel showing purified Rpr protein incubated at RT for different times. Right, cartoon of monomeric Rpr with known structural elements, as well as cartoon of Rpr dimer. (F) MALDI–ToF analysis of purified Rpr protein, which confirms the MW of Rpr and Rpr dimer. (G) Left, Coomassie-stained SDS-PAGE gel showing the apparent formation of Rpr–Thsp adducts following incubation of Rpr with Thsp, in a time-dependent fashion. Right, schematic model of Rpr–Thsp adduct and of Thsp bridging between two Rpr molecules, (Rpr)_2_-Thsp. (H) MALDI–ToF confirmation of Rpr–Thsp and (Rpr)_2_–Thsp adducts formation.

Given the presence of reactive DHA and DHB residues in Thsp and its reported ability to interact with proteins such as TipAS, we investigated whether Thsp inhibits protein degradation by interacting covalently with cellular proteins 17. Being a large molecule, the interaction with a protein might cause a shift in protein size that can be detected by WB. To test this hypothesis, we incubated extracts of HEK293 cells with either 75 μM or 7.5 μM Thsp for 1 hr or just briefly for 3 min., and tested for changes in these extracts by WB using available antibodies. We noticed that certain proteins such as p53 or Usp24 either show the formation of additional higher molecular bands or have undergone complete migration shifts (Fig.3A). This reaction appears to develop in time, as short incubation does not lead to detectable migration shifts (Fig.3A). To determine whether these effects occur in living cells, we treated HEK293 with Thsp in a time-dependent fashion and tested by WB the appearance of p53 higher molecular bands, as an indicator of Thsp (re)activity. This is indeed the case, as the formation of p53 higher molecular bands became distinct at 4–8 hrs treatment (Fig.3B). Appearance of bands consistent with dimers and oligomers was also observed for DIAP1 (Fig.3C) and Rpr (Fig.3D), which are expressed ectopically in HEK293 as part of the DIAP1 sensor.

Since Rpr appears to be modified by Thsp in cells, we decided to investigate this interaction *in vitro*, as a model of Thsp–protein interaction. Rpr has only 65 amino acids, which makes it easier to analyse by mass-spectrometry (MS) and contains a single Cysteine residue (Cys 49) that simplifies the possibilities of potential interactions with Thsp. As such, purified Rpr was examined by Coomassie staining, which confirmed its size and also the presence of protein dimers in small amounts (Fig.3E). Then, purified Rpr was analysed by MALDI-ToF MS, which confirmed the expected MW and the presence of Rpr dimers (Fig.3F). In parallel, purified Rpr was incubated with Thsp in a time-dependent fashion and the samples were analysed as above. Thiostrepton induced formation of novel bands besides Rpr, which are consistent with potential Rpr-Thsp and (Rpr)_2_-Thsp adducts by Coomassie staining (Fig.3G). Indeed, the formation of Rpr-Thsp and (Rpr)_2_-Thsp adducts species was confirmed by MALDI-ToF MS (Fig.3H). The existence of protein-Thsp adducts at 1:1 ratio has been previously demonstrated with *Streptomyces* protein TipAS, yet this is the first demonstration of a Protein–Thsp–Protein adduct (2Protein: 1Thsp ratio), which explains the appearance of protein dimers observed in human cells following Thsp treatment 17. Despite its abundance in SDS-PAGE, detection of the (Rpr)_2_-Thsp adduct by MALDI–ToF MS has been difficult, and only solubilization of the sample in 50% formic acid has enabled its unequivocal detection (Fig.3H).

### Covalent binding of Thiostrepton to proteins blocks proteasome function

**Figure 4:**
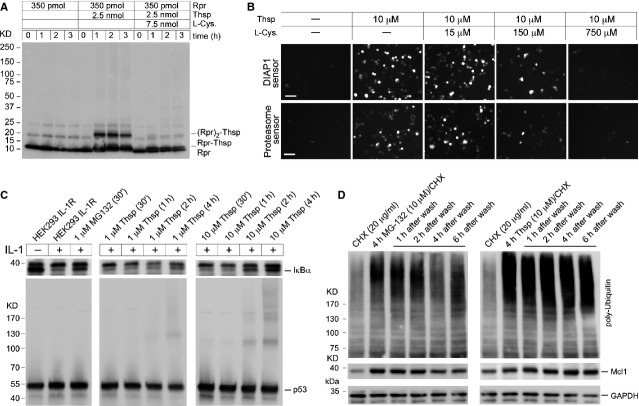
Proteasome inhibition is dependent on Thiostrepton ability to bind covalently to proteins. (A) Time-dependent Rpr–Thsp adducts formation is prevented by L-Cysteine supplementation. Rpr was incubated *in vitro* with Thsp or Thsp/L-Cysteine combination and the formation of Rpr adducts was monitored by WB with Rpr-specific antibody. This is consistent with Thsp binding covalently to the Cysteine residue of Rpr (Cys49), a process that is blocked by addition of excess L-Cysteine. (B) Microscopic images showing the fluorescence response of DIAP1 sensor cells and proteasome sensors cells following treatment with Thsp or combination Thsp/L-Cysteine. Excess L-Cysteine blocks Thsp-induced accumulation in sensors’ fluorescence (or blocks Thsp-induced proteasome inhibition), which is consistent with a model where Thsp covalent attachment to Cysteine residues is determinant for its proteasome inhibitory activity. (C) Thsp is a slow inhibitor of the proteasome activity in the cell when compared to chymotrypsin activity inhibitor MG-132, and the proteasome inhibition by Thsp correlates with formation of Thsp–p53 adducts in the cell. HEK293 IL-1R cells were treated with MG-132 or Thsp followed by stimulation with IL-1 (10 ng/ml) for 15 min., to trigger proteasomal degradation of IκBα (NF-κB activation). Persistence of IκBα in samples pre-treated with MG-132 or Thsp and subsequently with IL-1, is an indication of proteasome inhibition. IκBα stabilization in Thsp treated samples correlates with accumulation of p53 multimeric bands (an indicator of Thsp–protein adducts formation). (D) WB detection of polyubiquitination and Mcl1 accumulation in extracts of Raji cells. The cells were treated with CHX, CHX/MG-132 and CHX/Thsp for 4 hrs, washed and supplemented with fresh media and then harvested at different time-points (1–6 hrs). GAPDH was used as a loading control. The extent of polyubiquitination and Mcl-1 levels, 4 hrs after washing off MG-132 are largely reversed to non-treated state. For comparison, these effects remained steady or increased 4 hrs after Thsp wash off, which is consistent with a covalent-binding proteasome inhibition model.

Thiostrepton can interact covalently with Rpr. Next, we showed that the formation of Rpr-Thsp adducts *in vitro* is counteracted by the supplementation of the reaction with excess L-Cysteine (Fig.4A). This is an indication that Thsp interacts with Cys49 in Rpr, as predicted. Furthermore, we found that the ability of Thsp to block proteasome function in human cells (as visualized by fluorescence accumulation in DIAP1 and proteasome sensor cells) is quenched by the addition of L-Cysteine (Fig.4B). This suggests that Thsp needs to bind covalently to Cysteine residues to block the proteasome function. Subsequently, we asked whether Thsp is a rapid or slow inhibitor of proteasome function in the cell. To this end, we followed the proteasome-mediated IκBα degradation in HEK293 interleukin (IL)-1R cells, pre-treated with MG-132 or Thsp and then activated with IL-1 (Fig.4C). Interleukin-1 treatment leads to IκBα degradation as part of signalling leading to NF-κB activation 39. Thirty minute preincubation of cells with MG-132 is sufficient to prevent IκBα degradation (Fig.4C). By comparison, Thsp achieves the same effect only after 4 hrs of pre-incubation and at a concentration 10-fold higher than MG-132 (Fig.4C). Interestingly, the slow rate of proteasome function impairment by Thsp, correlates with the appearance of p53-Thsp adducts (Fig.4C, lower panel) and is in agreement with previous observations of Thsp–protein adducts which are formed in a time-dependent fashion (Fig.3A). Covalent attachment to proteins and slow proteasome inhibition, suggest the possibility that Thsp is an irreversible inhibitor of proteasome function. To address this question, we treated Raji cells with CHX (to prevent *de novo* protein production), CHX/MG-132 or CHX/Thsp combinations. Four hours after treatment, the drugs were washed off the cells and the cells were further incubated for 1–6 hrs. As markers for proteasome function we monitored the ubiquitination status in cell extracts and the levels of labile Mcl-1 protein. MG-132 treatment for 4 hrs induced the accumulation of polyubiquitinated species and Mcl-1 as expected. However, these effects were reversed almost completely 4 hrs after MG-132 withdrawal, which indicates that the proteasome function was restored in these cells (Fig.4D). When Raji cells were incubated with Thsp for 4 hrs, we observed the accumulation of polyubiquitinated species and a slight increase in Mcl-1. However, 4 hrs after Thsp withdrawal, the polyubiquitination remained strong and Mcl-1 levels continued to increase, which is in contrast to MG-132 (Fig.4D). Collectively, these observations are consistent with an irreversible action of Thsp on proteasome function in the cell.

### Thiostrepton attached to substrates precludes substrate degradation by the proteasome

**Figure 5:**
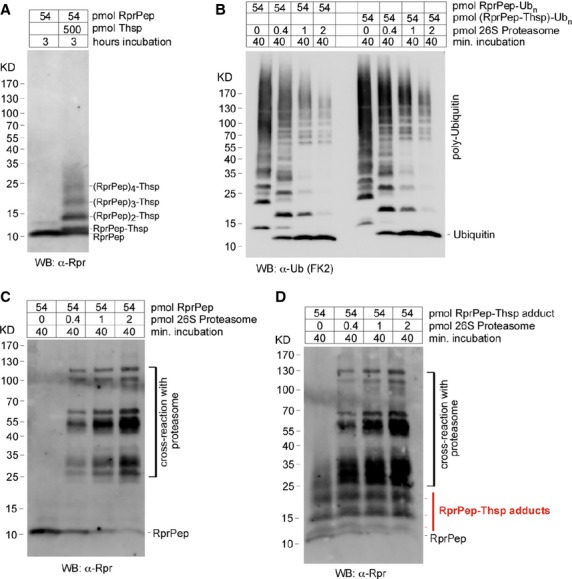
Proteasome substrate covalently modified by Thiostrepton is not degraded by 26S proteasome. (A) WB detection of a 37 amino acids chimeric Rpr peptide (RprPep) and RprPep-Thsp adducts formed *in vitro*, using a Rpr-specific antibody. (B) WB detection of purified 26S proteasome mediated deubiquitination of *in vitro* polyubiquitinated RprPep or RprPep-Thsp adducts. Thiostrepton covalently attached to a polyubiquitinated substrate does not interfere with the ability of 26S proteasome to deubiquitinate substrates. (C) WB detection of non-ubiquitinated RprPep degradation by purified 26S proteasome. (D) WB detection of non-ubiquitinated RprPep-Thsp adducts degradation by purified 26S proteasome. Thiostrepton modified RprPep is not degraded by 26S proteasome *in vitro*.

To understand how Thsp-modified substrates are processed by the proteasome, we needed a substrate that can be modified by Thsp and that can be polyubiquitinated *in vitro*. Thus, we generated RprPep (Fig. S1) that is based on Rpr protein, in which the folded central helical region of Rpr was replaced with an HA tag, to generate an essentially unfolded peptide. *In vitro* incubation with Thsp, led to efficient formation of RprPep-Thsp adducts (Fig.5A). RprPep being smaller than Rpr and unfolded, allowed the formation of additional oligomeric adducts (trimers, tetramers). We have previously established an *in vitro* assay for Rpr ubiquitination that could be used for RprPep ubiquitination as well 29. As such, we have ubiquitinated *in vitro* RprPep and RprPep-Thsp adducts samples, and incubated them *in vitro* with purified 26S proteasomes to determine how these samples are deubiquitinated. Interestingly, incubation with increasing amounts of 26S proteasome, showed a very similar deubiquitination pattern (Fig.5B). This indicates that Thsp-modified substrates do not impair the ability of the proteasome to deubiquitinate substrates. Next, we wanted to determine whether RprPep and the RprPep-Thsp adducts are degraded by the 26S proteasome. Incubation of RprPep with increasing amounts of 26S proteasome led to peptide degradation in a proteasome concentration manner (Fig.5C). Interestingly, RprPep-Thsp adducts are not degraded by 26S proteasome under the same conditions (Fig.5D), suggesting that the Thsp may hinder sterically the substrate/peptide translocation through the channels/gates of the proteasome.

### Thiostrepton interacts covalently with subunits of the 19S Base and proteasome substrates

**Figure 6:**
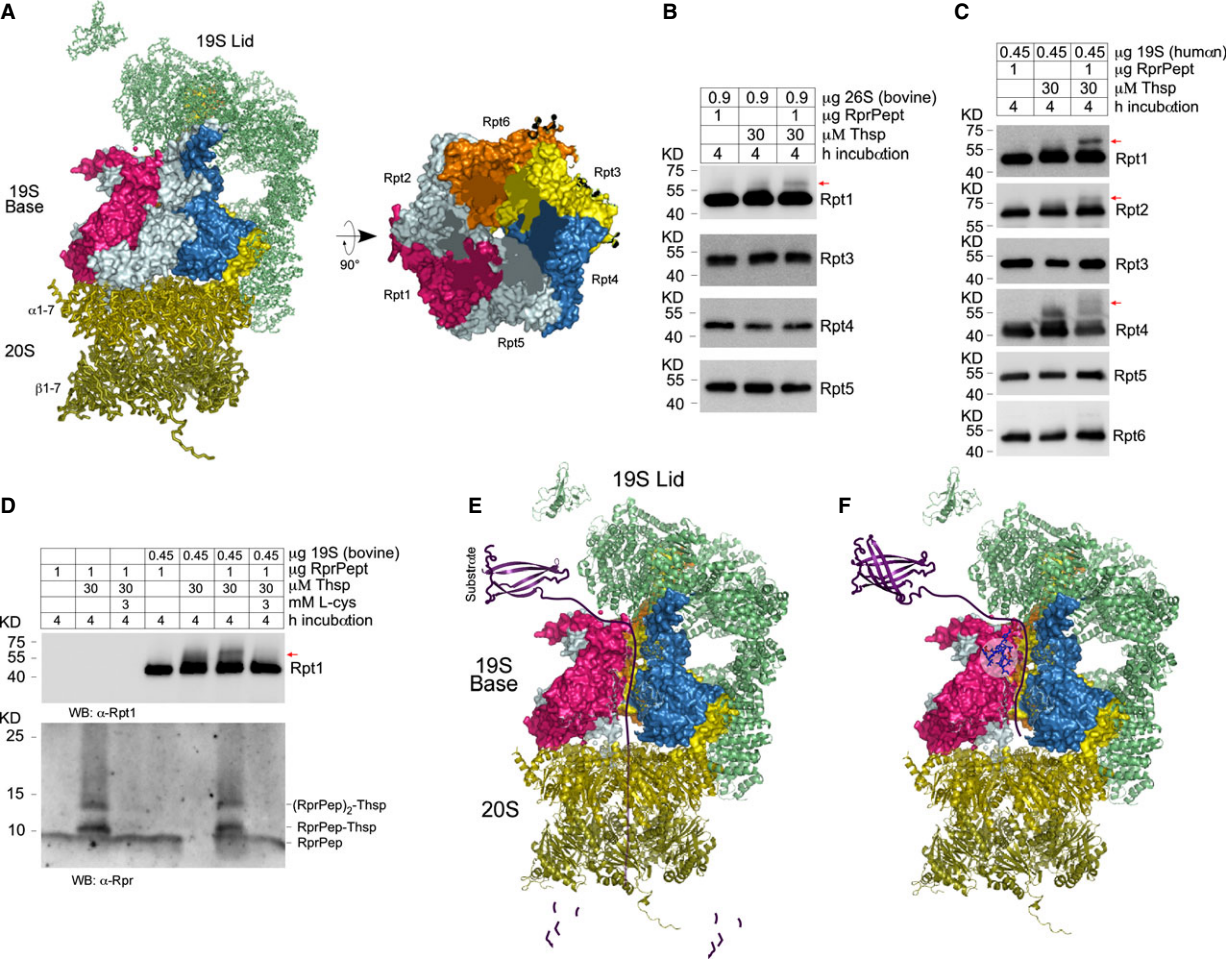
Thiostrepton links protein substrates covalently to the 19S subunit of the proteasome. (A) Left, representation of the yeast 26S proteasome structure, based on reported PBD code: 4B4T. The 19S subunit consists of the Lid (top, green semi-transparent) and the Base (middle, multicolour). The 20S subunit (on the bottom, yellow semi-transparent) entails the proteolytic sites and represents a platform for 19S attachment. Right, top view representation of the 19S Base, with each of the 6 Rpt subunits distinctly coloured and labelled. (B) WB detection of Rpt1, Rpt3, Rpt4 and Rpt5 following incubation of bovine 26S proteasome with RprPep, Thsp or both. The data show the appearance of a new band above Rpt1, in samples containing 26S, RprPep and Thsp, which indicates that RprPep is linked covalently to the Rpt1 subunit *via* Thsp. (C) WB detection of Rpt1, Rpt2, Rpt3, Rpt4, Rpt5 and Rpt6 in assays containing human 19S regulatory particle incubated with RprPep, Thsp or both. Appearance of new bands indicates that Thsp attaches RprPep covalently to Rpt1 primarily, and weakly to other base subunits such as Rpt2 and Rpt4. (D) WB detection of Thsp adduct formation with RprPep and Rpt1 in the context of bovine 19S proteasome. Addition of excess L-cysteine blocks formation of RprPep–Thsp and that of a distinctive band above Rpt1 (red arrow), which is consistent with an Rpt1–Thsp–RprPep adduct. (E) Cartoon model of substrate degradation by 26S proteasome, which include deubiquitination mediated by the 19S Lid, substrate unfolding and translocation (mediated by the 19S Base) and subsequent substrate degradation (mediated by 20S). (F) Model of Thsp-induced proteasome inhibition, with Thsp binding to Rpt1 subunit and blocking the process of substrate unfolding and translocation. During this process, proteasome substrates may get covalently attached to Rpt subunits as well. Thiostrepton is shown in blue and is highlighted by a semi-transparent circle.

A biotinylated Thsp derivative was used previously to pull down binding partners in extracts of human cells 21. Among the many potential candidates identified by MS there were several proteasome subunits. A closer inspection indicated that many of those proteins were Rpt subunits of the Base in the 19S proteasome regulatory particle (Fig.6A). To address the potential Thsp interaction with Rpt proteasome subunits, we incubated purified bovine 26S proteasome with Thsp, RprPep or its combination and tested it by WB to verify whether any subunits of the Base (Rpt proteins) were modified. Remarkably, from the Rpt subunits tested Rpt1 showed a clear protein shift that was formed only in the presence of Thsp and RprPep, which can only be explained by the formation of a Rpt1–Thsp–RprPep adduct (Fig.6B). To confirm this discovery, we next tested the ability of Thsp to form adducts with the human 19S proteasome and the RprPep substrate. To this end, purified human 19S was incubated as above with Thsp, RprPep or its combination and the formation of adducts with Rpt subunits was tested by WB. Following the testing of all Rpt subunits of the Base and consistently with the findings in panel B, Thsp attaches the proteasome substrate RprPep primarily to Rpt1 subunit of the 19S regulatory particle. Additional Rpt proteins such as Rpt2 and 4 serve as weaker binding partners (Fig.6C). Next, we investigated whether Rpt1–Thsp–RprPep adduct formation can be quenched by the supplementation of the reaction with excess L-cysteine. For this purpose, we incubated bovine 19S proteasome with Thsp, RprPep or both in the absence or presence of excess L-cysteine (Fig.6D). Consistent with observation in panels B and C we saw the appearance of a band above Rpt1 only in the presence of Thsp and RprPep, suggesting the formation of Rpt1–Thsp–RprPep adducts. Interestingly the addition of excess L-cysteine prevented the formation of this band, which strongly correlates with the inability of Thsp to interact with cysteine residues in Rpt1 and RprPep. The quenching effects of cysteine are specific, as it blocked the formation of RprPep–Thsp adducts in the absence or presence of bovine 19S (Fig.6D). These data are consistent with a model where Thsp blocks the proteasome function by interacting with the Rpt subunits of the 19S proteasome, and potentially by covalent attachment of proteasome substrates to the Base of the 19S proteasome, during the process of substrate unfolding and translocation (Fig.6E and F).

## Discussion

We identified Thsp as a strong inhibitor of DIAP1 degradation in a high-content high-throughput screen. Thiostrepton inhibits UPP-mediated protein degradation in a variety of assays, including a cell-based proteasome reporter, accumulation of polyubiquitinated proteins, stabilization of DIAP1, Rpr, Mcl-1 and p21. Previous *in vitro* studies using purified 20S proteasomes and synthetic small peptide-like substrates suggested that Thsp is a chymotrypsin inhibitor of the proteasome 14,22,23. However, in contrast to its effects on chymotrypsin activity, Thsp activated the trypsin-like activity of 20S proteasome, while the caspase-like activity remained largely unaffected. In addition, Thsp did not block the chymotrypsin activity when using purified 26S proteasomes, unlike the chymotrypsin inhibitor MG-132. Therefore, we investigated the mechanism by which Thsp blocks the proteasome activity in more detail. We suggested that the ability of Thsp to prevent protein degradation *in vivo* may be related to both its large size (1.66 kD) and ability to bind covalently to cellular proteins. An inspection of the tri-dimensional structure shows that Thsp is 26 Å long and 18.2 Å wide 40 (Fig. S2A). The diameter of the 20S proteasome gate in open conformation is approximately 22.5 Å towards the outside and 16.7 Å towards the inside 41 (Fig. S2B). Therefore, Thsp is slightly larger in size than the 20S proteasome gate and presumably cannot enter the 20S proteasome. One hypothesis is that Thsp may block protein breakdown by preventing the entry of substrates that are covalently linked to this compound. To test this model, we used a 37 amino acid chimeric Rpr peptide (RprPep) that can be covalently linked to Thsp and ubiquitinated. Linkage of Thsp to this substrate did not interfere with deubiquitination, but it specifically rendered it resistant to proteasomal degradation. This is consistent with the idea that Thsp sterically prevents the substrates it is attached to from translocating through proteasome pores.

Thiostrepton contains reactive DHA and DHB residues and is known to form adducts with the *Streptomyces* TipAS protein at 1:1 ratio (TipAS-Thsp) 17. Here, we showed that proteins in human cells including p53, Usp24 or *Drosophila* proteins DIAP1 and Rpr are modified by Thsp to trigger the formation of apparent dimers and other oligomeric species, as it was reported for Peroxyredoxin-3 16. Importantly, this report is the first demonstration of a protein–Thsp adduct at 2:1 ratio (Rpr_2_-Thsp). Our MS data clarifiy the nature of dimeric species that are observed in cells, as being protein dimers bridged by Thsp (Fig.3H). Despite DHA and DHB propensity to interact with cysteine residues, we find that Thsp does not interact with many cysteine-containing proteins (not shown) and is not an unspecific DUB inhibitor as it does not outcompete a DUB specific probe (HA-Ubiquitin-VME) from interacting covalently with the cysteine residue in the active site of human DUBs (Fig. S3) 42. These results argue for a defined selectivity of Thsp in substrate addition.

The ability of cysteine supplementation to quench the formation of Protein–Thsp adducts as well as to restore the proteasome function in cells is consistent with the notion that Thsp needs to attach covalently to proteins to inhibit proteasome function. Furthermore, the slow rate of proteasome inhibition by Thsp andthe persistence of proteasome inhibition markers after Thsp withdrawal, indicate that Thsp is an irreversible inhibitor of proteasome function in cells. The proteasome chymotrypsin-like activity inhibitor Velcade/Bortezomib is currently used in the clinic for the treatment of multiple myeloma and mantle cell lymphoma. However, the development of resistance that is often observed following therapy suggests that novel approaches of proteasome inhibition, including the 19S regulatory particle, might be needed 25.

A Thsp probe was previously used to pull down protein binding partners from a human cell extract and revealed several proteasomal proteins among a long list of potential interacting partners 21. A closer inspection indicates that these proteins are subunits of the Base of the 19S proteasome regulatory particle. The 19S Base consists of 6 Rpt proteins (1–6) organized as heterodimers, which form a cylinder with a central pore and anchors on top of the 20S particle 43,44. The Rpt proteins are ATP-ases and function in substrate unfolding – translocation step – which occurs after substrate deubiquitination. Interestingly, we discovered that Thsp is able to interact covalently with proteasome Base subunits (primarily Rpt1) and the proteasome substrate (RprPep), in the context of bovine 26S proteasome and human 19S regulatory particle. These data provide direct evidence for the interaction between Thsp and Rpt subunits of the 19S proteasome, but also for the presence of proteasome–proteasome substrates covalent adducts. Because only a fraction of Rpt1 was covalently linked to RprPep substrate, this cannot explain the extent of proteasome inhibition by Thsp in the cells. However, these experiments cannot rule out that a larger fraction of Rpt1 is actually modified by Thsp, but Thsp alone may not trigger a visible shift by western blot (Rpt1 is a large protein, 50 kD). Taken together, our findings are consistent with a model of irreversible 19S proteasome inhibition by Thsp. This is a novel mechanism of proteasome inhibition by a natural product, which forms a covalent adducts with 19S proteasomal subunits and proteasome substrates, therefore blocking the process of substrate unfolding and translocation.

## Supporting Information

**Data S1** Model for formation of RprPep-Thsp adducts.
