# Supplementary material for: Thiostrepton interacts covalently with Rpt subunits of the 19S proteasome and proteasome substrates
Source: J Cell Mol Med. 2015 May 30;19(9):2181–92. doi: 10.1111/jcmm.12602 (PMC4568923; doi:10.1111/jcmm.12602)
Supplement: Supplementary file 1 [file jcmm0019-2181-sd1.pdf]

Supplementary Information

Thiostrepton Interacts Covalently with Rpt Subunits of the 19S Proteasome and Proteasome Substrates

Cristinel Sandu[1], Nagaranjan Chandramouli[2], J. Fraser Glickman[3], Henrik Molina[2], Chueh-Ling, Kuo[4], Nikolay Kukushkin[4], Alfred L. Goldberg[4] and Hermann Steller[1,*]

[1]Howard Hughes Medical Institute, Strang Laboratory of Apoptosis and Cancer Biology, The Rockefeller University, New York, NY, 10065. [2]Proteomics Resource Center, The Rockefeller University, New York, NY, 10065. [3]High Throughput Screening Resource Center, The Rockefeller University, New York, NY, 10065.[4]Department of Cell Biology, Harvard Medical School, Boston, MA, 02115.

[*]Correspondence to:

Dr. Hermann Steller

Laboratory of Apoptosis and Cancer Biology,

HHMI/The Rockefeller University.

1230 York Avenue, Box 252, New York, NY, 10065.

Tel: 212-327-7075

Fax: 212-327-7076

Email: steller@rockefeller.edu

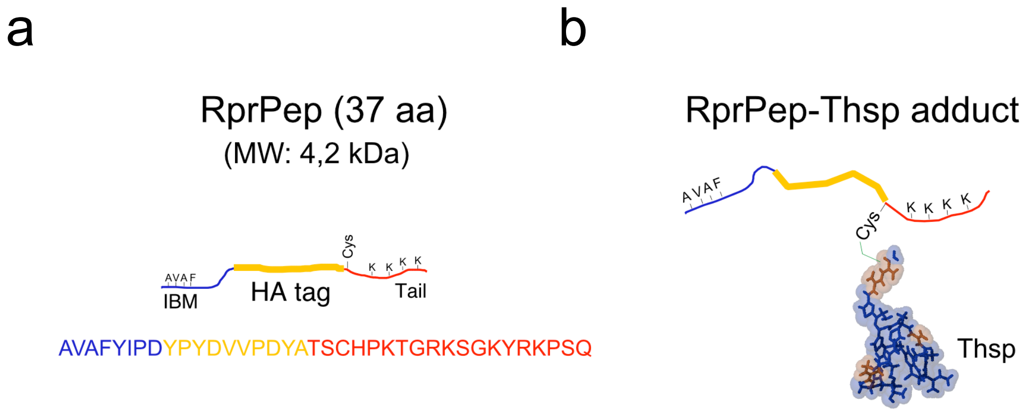

Supplementary Figure 1. **Model for formation of RprPep-Thsp adducts.** (**a**) Representation of RprPep features including MW, structural elements and the amino acid sequence. (**b**) Schematic representation of an adduct between Thsp and the only Cysteine residue in RprPep.

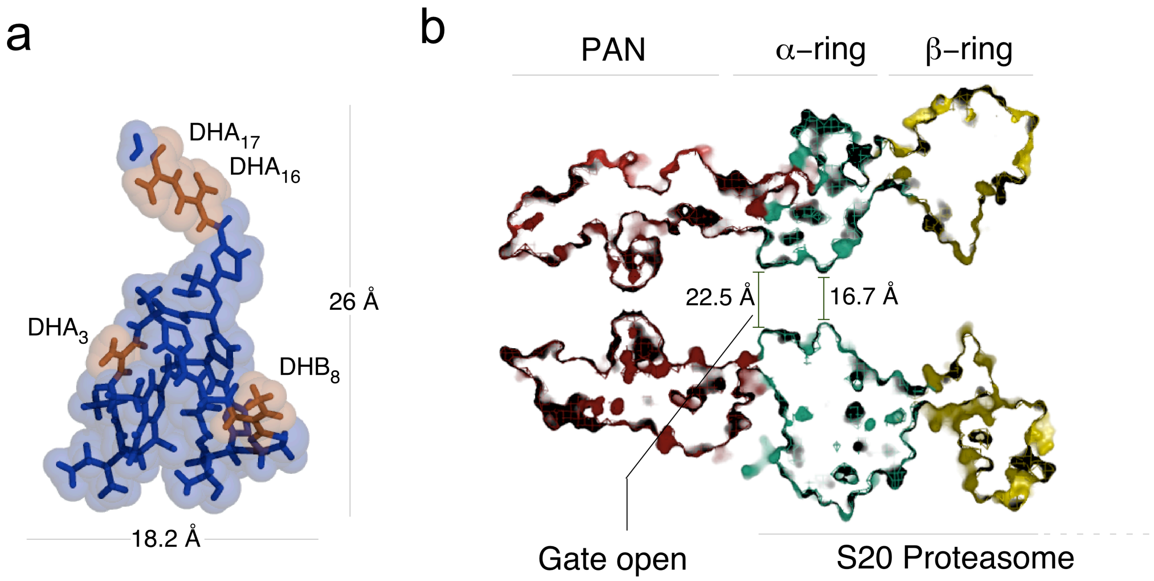

Supplementary Figure 2. **Overview on the size of Thiostrepton and "open gate" conformation of the 20S proteasome**. (**a**) Representation of Thsp (PDB: 2L2W) three-dimensional structure. Size measurement on x- and y-axes are represented in angstroms (Å). Dehydroalanine (DHA) and dehydrobutyrine (DHB) reactive groups are marked in red. (**b**) Cross section of the archaeal 20S proteasome bound to PAN (PDB: 3IPM). The PAN ATPase ring complex is shown in red, while the 20S subunit (symmetric half) is shown in green (α-ring) and yellow (β-ring). The cross-section shows the 20S proteasome gate in open configuration and the diameter of the pore on the outside (22.5 Å) as well as on the internal side of α-ring (16.7 Å).

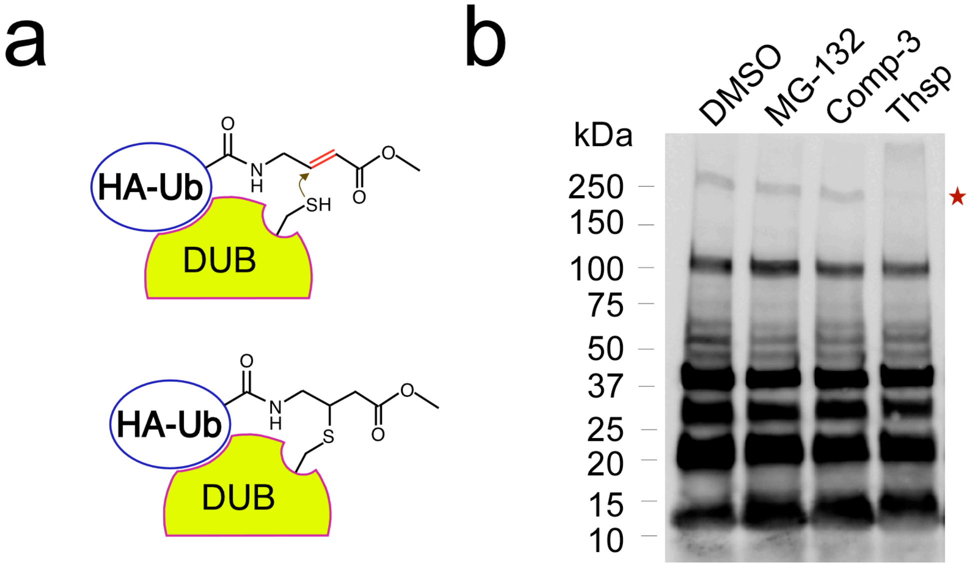

Supplementary Figure 3. **Effect of Thiostrepton on Ub-VME coupling on human DUBs.** (**a**) Schematic representation of an adduct formation between vinyl-methyl ester (VME) in HA-Ub-VME probe and the reactive cysteine residue in human DUBs. Ubiquitin interacts with DUBs and brings the VME moiety in the vicinity of the reactive cysteine present in the active site of several families of human DUBs. The unsaturated α,β-carbonyl group of VME (marked in red) reacts with the thiol group of the cysteine residue via a Michael addition reaction, and leads to formation of a strong covalent C-S bond between HA-Ub-VME and human DUBs. (**b**) Effect of DMSO, MG-132, Comp-3, and Thsp on covalent binding of HA-Ub-VME to DUBs present in HEK293 cell extracts. DUBs covalently coupled with HA-UB-VME are detected by western blot using an anti-HA antibody. Red star shows the lack of a DUB labeling (250 kDa) in sample treated with Thsp.

Methods

In vitro labeling of DUBs with HA-UB-VME. HA-Ub-VME was purchased from Enzo Life Sciences. The assay consisted of 40 μl reaction that contained 25 μg HEK293 cell extract (in lysis buffer described in Methods section), 1 μM HA-Ub-VME, 5 mM $MgCl_2$, 10 mM ATP and compounds (MG-132, Comp-3 and Thsp) at a final concentration of 75 μM. Reaction buffer was 25 mM Tris, pH 7.4, 50 mM NaCl, 250 μM DDT. The reaction was incubated at 37 °C for 1 hour, then supplemented with 10 μl SDS-PAGE sample buffer and boiled for 5 minutes at 95 °C. 10 μl of the sample was then separated by SDS-PAGE, followed by western blot detection of UB-VME labeled proteins with an HA antibody.
